# Transcriptome Analysis of Eggplant Root in Response to Root-Knot Nematode Infection

**DOI:** 10.3390/pathogens10040470

**Published:** 2021-04-13

**Authors:** Min Zhang, Hongyuan Zhang, Jie Tan, Shuping Huang, Xia Chen, Daohong Jiang, Xueqiong Xiao

**Affiliations:** 1 State Key Laboratory of Agricultural Microbiology, Hubei Key Laboratory of Plant Pathology, College of Plant Science and Technology, Huazhong Agricultural University, Wuhan 430070, China; zhangmin818@aliyun.com (M.Z.); daohongjiang@mail.hzau.edu.cn (D.J.); 2 Wuhan Academy of Agricultural Sciences, Wuhan 430065, China; zhanghongyuan@whu.edu.cn (H.Z.); aggas@126.com (J.T.); hsping80@163.com (S.H.); chenxia_001@126.com (X.C.)

**Keywords:** eggplant, *Solanum torvum*, RNA-seq, root-knot nematode, ABA, resistance

## Abstract

Eggplant (*Solanum melongena* L.), which belongs to the Solanaceae family, is an important vegetable crop. However, its production is severely threatened by root-knot nematodes (RKNs) in many countries. *Solanum torvum*, a wild relative of eggplant, is employed worldwide as rootstock for eggplant cultivation due to its resistance to soil-borne diseases such as RKNs. In this study, to identify the RKN defense mechanisms, the transcriptomic profiles of eggplant and *Solanum torvum* were compared. A total of 5360 differentially expressed genes (DEGs) were identified for the response to RKN infection. Gene Ontology and Kyoto Encyclopedia of Genes and Genomes enrichment analysis showed that these DEGs are mainly involved in the processes of response to stimulus, protein phosphorylation, hormone signal transduction, and plant-pathogen interaction pathways. Many phytohormone-related genes and transcription factors (MYB, WRKY, and NAC) were differentially expressed at the four time points (ck, 7, 14, and 28 days post-infection). The abscisic acid signaling pathway might be involved in plant-nematode interactions. qRT-PCR validated the expression levels of some of the DEGs in eggplant. These findings demonstrate the nematode-induced expression profiles and provide some insights into the nematode resistance mechanism in eggplant.

## 1. Introduction

Eggplant (*Solanum melongena* L.) is an important solanum genus vegetable crop, originating in India, and now widely grown in Asia, Africa, and the Mediterranean [1]. In 2016, 46.63 million tons of eggplant were produced in the top three producing countries: China (32.03 million tons), India (12.55 million tons), and Egypt (1.19 million tons), according to the Food and Agriculture Organization of the United Nations (FAO; http://faostat.fao.org, accessed on 1 October 2016). It has been reported that eggplant is susceptible to several bacterial and fungal pathogens, such as the *Verticillium dahlia*, fungus, and nematodes [2,3]. Root-knot nematodes (RKNs, Meloidogyne spp.) are devastating endoparasites that parasitize many cultivated plants and seriously threaten global food safety. The estimated crop globally losses due to RKN infections are approximate USD 157 billion annually. It parasitizes a variety of cultivated plants, including tomato (*Solanum lycopersicum*), carrot (*Daucus carota*), potato (*Solanum tuberosum*), eggplant (*Solanum melongena*), and watermelon (*Citrullus vulgaris*) [4,5]. 

RKNs are among the most damaging plant pathogens that reduce yield. Once infected, large knots are formed on the roots of host plants. They ultimately result in giant cells, which provide fewer resources to the surrounding root tissues [6,7]. The southern root-knot nematode *Meloidogyne incognita* is one of the most sophisticated plant-parasitic nematodes in China. It is a sedentary endoparasite that completes its lifecycle inside the host roots in 4–6 weeks. *M. incognita* invades the plant roots at the second stage juveniles (J2s) and induces normal root cells to dedifferentiate into giant cells in its feeding site [8,9]. The giant cells are a kind of multinucleate and hypertrophied cells, which serve as a source of nutrition for growing nematodes. The J2s become sedentary and undergo a few rounds of molting to J3s, J4s, and adults. Then, adult female nematodes lay the eggs on the surface of the root and, following the hatch, generate infectious J2s, which start their next parasitic life cycle [9,10]. 

When the RKNs invade the plants, they can respond with a succession of defense reactions. Several phytohormones, including auxin, cytokinins, salicylic acid, jasmonate, gibberellin, abscisic acid, and brassinosteroids (BRs), play important roles in response to abiotic stress. Auxin and cytokinin have been reported to modulate plant immune response pathways in plant-nematode interactions [11]. In Arabidopsis, salicylic acid-related genes were induced in RKN-infected roots [12]. In addition, transcription factors participate in response to RKN infection. WRKY is a class of transcription factors that are widely involved in the response to nematode infection. In pepper, WRKY2 was reported to be induced during incompatible interactions of host plants with RKNs [13]. AtWRKY23 is an auxin-inducible gene that is expressed during the early stages of feeding site establishment [14].

*Solanum torvum* Sw., commonly known as turkey berry, is a wild relative of eggplant, widely cultivated in tropical and subtropical countries [15]. Turkey berry is resistant to RKNs and the majority of serious soil-borne diseases, providing genetic resources for improving disease resistance in eggplant. In addition, the rootstock of turkey berry is also used for grafting in eggplant cultivation. 

Previous studies have reported that plant-nematode interactions result in gene expression changes in the host plant [16]. Transcriptome analysis, especially RNA-Seq, has been widely employed to determine transcriptional changes in many plants [16,17]. The new and potent technique known as transcriptome analysis has been used to study susceptible or resistant plants during host-RKN interactions. Bagnaresi et al. (2013) combined 454 pyrosequencing and microarray technology to research the transcriptome profiling of *Solanum torvum* inoculated with the nematode *M. incognita* [18]. RKN-infected root samples resulted in 390 differentially expressed genes (DEGs) and enhancement of several processes, such as chitin catabolism and sesquiterpenoids biosynthesis. Shukla et al. (2018) used RNA-Seq data to reveal the gene expression profiles and metabolic networks during nematode responses in tomato [19]. In rice, transcriptional analysis of galls at 3 days after an infection with *Meloidogyne graminicola* (Mg) revealed that many DEGs are involved in the plant hormone biosynthesis and signaling pathways [20]. In many crops, the genetic mechanism of resistance to RKN has been preliminarily identified and characterized [4]. However, few studies have been published on the genetic mechanisms of RKN resistance in eggplant. 

In this study, the transcriptome profiles of RKN-infected eggplants root in *Solanum torvum* (RKN-resistant) and *Solanum melongena* (RKN-susceptible) were analyzed. Differentially expressed genes were identified in eggplants following *M. incognita* inoculation. Our results provide important insights into the underlying mechanism of the eggplant response to *M. incognita*, which provides a basis for further research.

## 2. Results

### 2.1. Analysis of RKN Infection in Eggplant Roots

**Figure 1:**
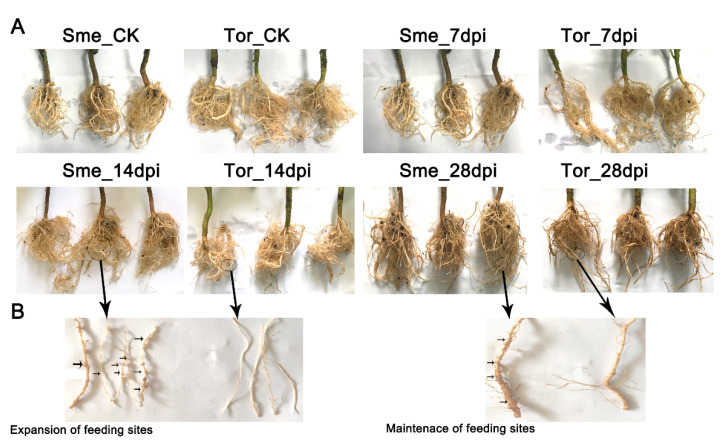
Morphological changes of roots infected by root-knot nematodes (RKNs) at different stages. (**A**) The different root morphologies between Sme and Tor in CK and at 7, 14, and 28 days post infection (dpi). (**B**) Comparison of the expansion feeding sites and maintenance feeding sites between ‘Sme’ and ‘Tor’. ‘Sme’ and ‘Tor’ stand for cultivated eggplant and wild eggplant, respectively.

After the RKN invasion, the number and diameter of root knots increased with the increase in inoculation days during the observation period (Figure 1A). A small amount of root nodes could be observed in cultivated eggplant (Sme), indicating that J2S had reached the feeding point and began to settle down at this stage (7 dpi), while root node formation was not observed in wild eggplant turkey berry (Tor). At 14 dpi, root knots were observed in the root tips of lateral roots at all levels of cultivated eggplant (Figure 1B), and some roots formed clusters, indicating that the cells at feeding sites began to form root knot galls around the enlarged giant cells (Figure 1B). The root growth of wild eggplant was normal, the taproot was obvious, and there were no obvious root knots in the root tip and the parts far from the root tip. At 28 dpi, the root nodes of cultivated eggplant seedlings continued to thicken and significantly increased, and piriform body appeared (Figure 1B).

### 2.2. Overview of RNA Sequencing

Twenty-four cDNA libraries were constructed from total RNA extracted from the torvum (Tor) and eggplant (Sme) roots (three replicates for each sample). The cDNA libraries were subjected to pair-end reading with the Illumina Hiseq 2500 platform, and generated 52–59 million and 67–87 million paired-end raw reads in Sme and Tor, respectively (Table S1). After removing low-quality reads and trimming adapter sequences using Trimmomatic, we obtained 50–58 million and 64–83 million clean reads for the Sme and Tor libraries, respectively. Three biological replicates were performed for each sample. Correlation analyses showed that the coefficient of the samples confirmed the consistency among the three biological replicates, indicating the test’s high reliability for further analyses (Figure S1). 

For Sme samples, we used the Eggplant Genome Database (http://eggplant.kazusa.or.jp/, accessed on 1 October 2014) as the reference genome. After quality control, the majority of the reads from Sme samples (83.16–91.18%) were successfully aligned to the reference genome (Table S1). Most of the reads were aligned to a single position. 

### 2.3. De Novo Assembly of Tor Transcriptome

Since the reference genome of Solanum torvum has not been reported, we here de novo assembled the transcriptome reads of the twelve Tor samples. In total, a range of 76.62–80.11% of the clean reads was mapped and assembled into contigs (Table S1). These contigs were then clustered into 152,489 transcripts and 78,228 unigenes (Table S2). The N50 values of transcripts and unigenes were 1942 and 1584 (Figure S2 and Table S2). The results were higher than the previous study of Turkey berry [21], which provided more information for further identification of the resistant genes in Tor (Turkey berry). Additionally, a total of 12,359 unigenes were annotated by the five databases using BLAST with E-value ≤ 1 × 10^−5^ (Figure S2). In addition, we identified 12,317 pairs of SSR primers which are valuable markers for genetic diversity analysis (Table S3). 

### 2.4. Identification of Differentially Expressed Genes under RKN Infection

**Figure 2:**
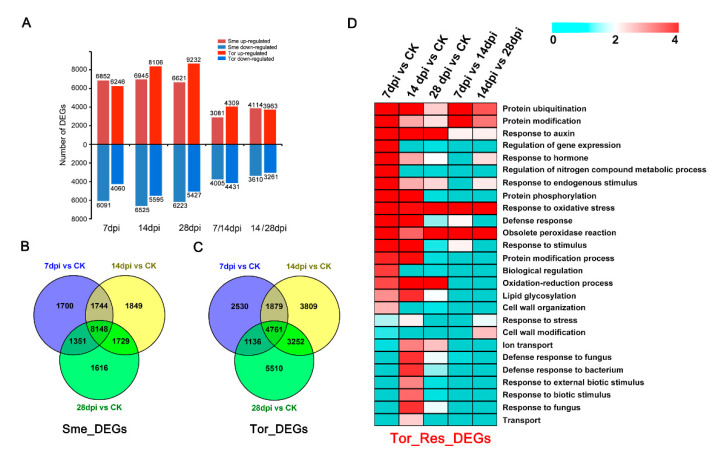
Differentially expressed genes (DEGs) of eggplant infected by root-knot nematodes (RKNs) with control (CK) and between the adjacent stages (7/14, and 14/28 dpi) in Sme and Tor. (**A**) The number of up- and down-regulated genes in Sme and Tor; (**B**) Venn diagrams showing the intersection of DEGs identified in Sme; (**C**) Venn diagrams showing the intersection of DEGs identified in Tor; (**D**) Gene Ontology (GO) enrichment analysis of the DEGs identified in Tor.

To investigate the response to RKN infections, the gene expression profiles at the three stages (7, 14, and 28 dpi) of Sme and Tor were analyzed. In total, 6852, 6945, and 6621 up-regulated DEGs and 6091, 6525, and 6223 down-regulated DEGs were identified in Sme compared with CK, respectively, while a total of 6246, 8106, 9232 DEGs, and 4060, 5595, and 5427 DEGs were up-regulated and down-regulated in Tor (Figure 2A). Compared with Sme, more up-regulated DEGs were identified in Tor at 14 dpi and 28 dpi (Figure 2A). A total of 8148 and 4761 DEGs were found to be common genes for the comparisons of three stages in Sme and Tor, respectively (Figure 2B,C). Gene Ontology (GO) enrichment analysis of the Tor responsive DEGs showed that the terms of response to oxidation stress, protein modification, response to stimulus, and signal transduction are significantly overrepresented (Figure 2D). 

### 2.5. Comparisons of Transcriptomes between Sme and Tor Responses to RKN Infection

**Figure 3:**
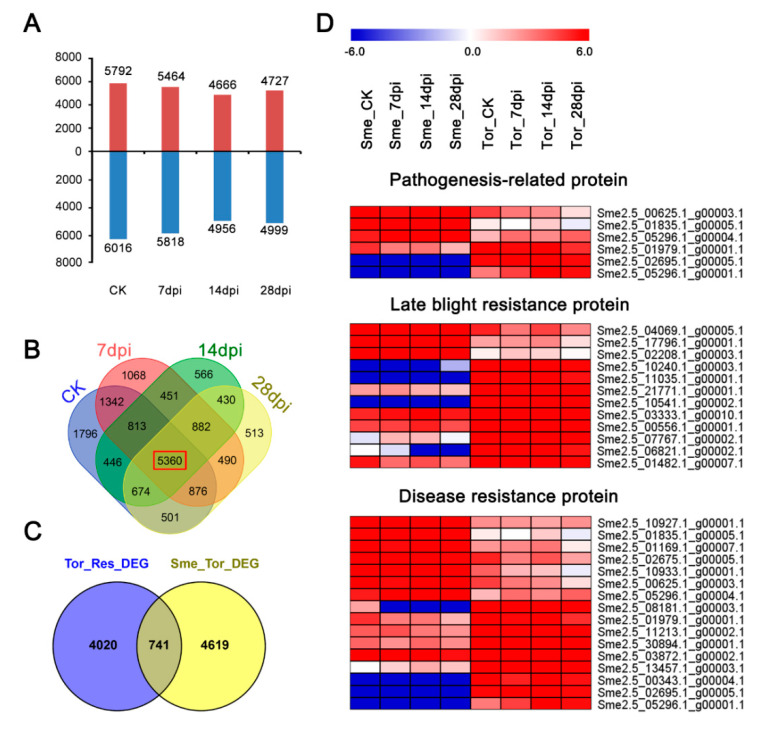
Differentially expressed genes (DEGs) of eggplant infected by RKNs at four stages between Sme and Tor and the expression patterns of disease resistance genes. (**A**) The number of up- and down-regulated genes between Sme and Tor at each stage (CK, 7, 14, and 28 dpi). (**B**) Venn diagrams showing the overlapping DEGs between Sme and Tor. (**C**) Overlap among RKN-responsive genes in Tor and DEGs between Sme and Tor. (**D**) Heatmaps showing the expression patterns of disease resistance genes, including pathogenesis-related, late blight resistance, and disease resistance genes.

To investigate the mechanisms of RKN invasion and gall formation, gene expression profiles between RKN-sensitive Sme and RKN-tolerant Tor were analyzed. Many DEGs were up- and down-regulated among the four stages between Sme and Tor (Figure 3A). A total of 5360 DEGs were shared by Sme and Tor during the four stages after RKN invasion, which could be the core genes involved in eggplant’s response to RKN (Figure 3B and Table S4). We found 741 DEG overlaps between the core genes and the DEGs identified in Tor, indicating these DEGs might be involved in the resistance to RKN infections (Figure 3C). We identified dozens of pathogen-related and disease-resistance genes that were differentially expressed during the four stages between Sme and Tor (Table S4). The DEGs included genes encoding the pathogenesis-related protein, late blight resistance protein, and disease resistance protein (Figure 3D). In addition, many *R* genes were detected, including NBS-LRR and RLK (LRR receptor-like kinases) (Figure 3D and Table S4). A total of 24 DEGs encoding *R* genes were identified with an NBS-LRR domain (Table 1 and Table S4). Most of these *R* genes were up-regulated in Tor among the three stages, but some of them were highly expressed in Sme compared with Tor (Table 1). For example, RGA4 (Sme2.5_10927.1_g00001.1), which encodes an NBS-LRR type R protein, was highly induced in all four stages (Table 1). 

### 2.6. Specific Differentially Expressed Gene in Tor Responses to RKN Infection

**Figure 4:**
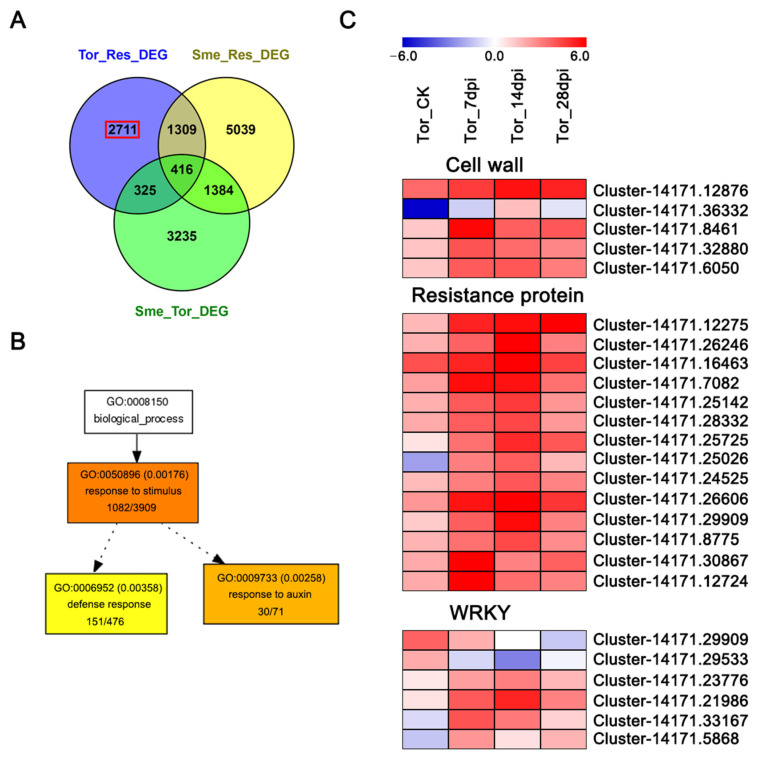
Tor-specific DEGs in responsive to RKNs. (**A**) Venn diagrams showing the specific responsive DEGs in Tor. (**B**) GO enrichment analysis the specific DEGs in Tor. (**C**) Heatmap showing the expression patterns of some specific DEGs in Tor.

Since *Solanum torvum* is a wild relative of eggplant, there may be some DEGs that were only involved in Tor in responsive to RKN infection. In our study, we identified 2711 DEGs were specific differentially expressed in the Tor (Figure 4A and Table S5). GO enrichment analysis also showed many of these DEGs were involved in ‘response to stimulus’, ‘defense response’ and ‘response to auxin’. The results indicate that more defense and resistance genes might be involved in the RKN infection of *Solanum torvum* than eggplant. The expression patterns of some specific DEGs in Tor revealed that these genes were up-regulated during the infection, including genes associated with the cell wall, resistance genes, and some WRKY transcription factors (Figure 4C). 

### 2.7. Analysis of Nematode-Responsive Hormone-Relative Genes and Transcription Factors

**Figure 5:**
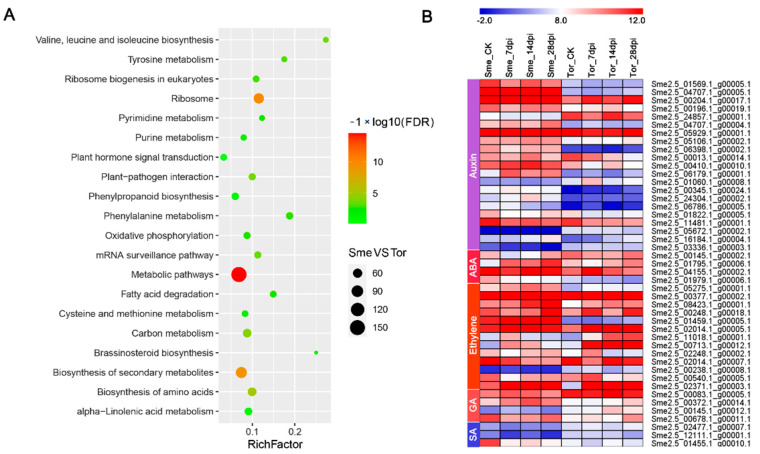
(**A**) The top 20 enriched Kyoto Encyclopedia of Genes and Genomes (KEGG) pathways for DEGs between Sme and Tor. Rich factor represents the ratio of the number of DEGs with the number of genes in the pathway. (**B**) Heatmap illustrating the DEGs related to phytohormones (auxin, abscisic acid (ABA), ethylene, GA and SA) between Sme and Tor.

KEGG pathway analysis revealed that the DEGs between Sme and Tor are involved in plant hormone signal transduction, plant–pathogen interaction, basal transcription factors, and phenylpropanoid biosynthesis (Figure 5A). Many of the DEGs are involved in the plant–pathogen interaction pathway, including CDPK, FLS2, RIN4, and WRKY (Figure S3 and Table 2). PR genes and WRKYs were highly expressed in Sme, which might participate in the phytoalexin accumulation and defense response (Table 2). Many of the genes encoding LRR receptor-like serine/threonine-protein kinase and TMV resistance protein were also up-regulated in Sme (Figure S4). However, eleven of the eighteen FLS2 (LRR receptor-like serine/threonine-protein kinase) genes were down-regulated in Sme (Table S4). In addition, some genes related to the cell wall were up-regulated in Sme, indicating these genes involved in the resistance to RKNs (Table 3).

Plant hormones are widely involved in the response to RKN infections in many plants [7]. In our study, we identified many DEGs related to phytohormones, such as auxin, abscisic acid (ABA), ethylene, gibberellic acid (GA), salicylic acid (SA), cytokinin, and brassinosteroid (BR) (Figure 5 and Table S6). Most of the auxin- and ethylene-associated genes were up-regulated in Sme (Figure 5B). However, many ABA-related genes were induced by RKN infection in Tor, such as 9-cis-epoxycarotenoid dioxygenase (NCED) and PYL8 (Figure 5A). PYL genes have been reported to be abscisic acid receptors, which play important roles in the resistance to stress [22]. The expression patterns of genes involved in ABA metabolism pathway under RKN infection were investigated. The *NCED* gene showed higher expression level in Tor than in Sme at all four stages. The other five ABA metabolism genes also showed differential expression between Sme and Tor (Figure S5). Two homologs of brassinosteroid-insensitive-1-associated receptor kinase (BAK1) (Sme2.5_03931.1_g00001.1, Sme2.5_03931.1_g00003.1), which are BR biosynthesis genes, were up-regulated in the Tor (Table S5).

**Figure 6:**
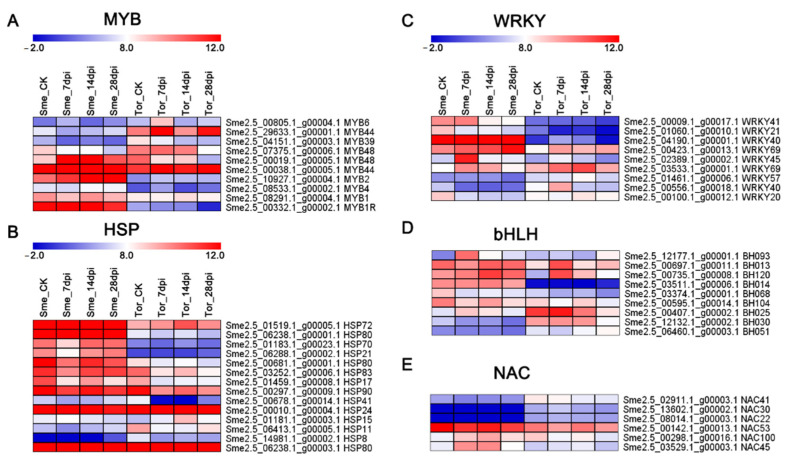
Heatmap showing the DEGs of transcription factors after infection by RKNs: (**A**) MYB, (**B**) HSP, (**C**) ERF, (**D**) WRKY, and (**E**) NAC.

**Figure 7:**
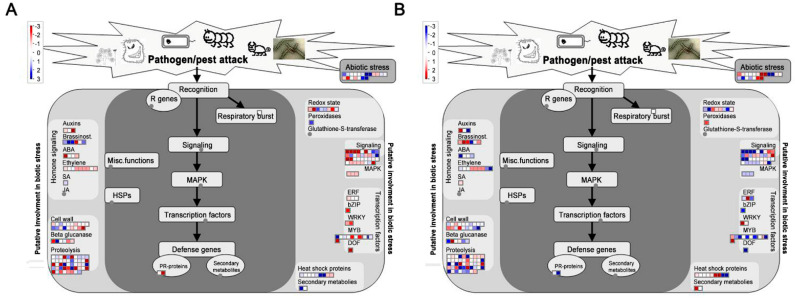
Overview of the DEGs of eggplant roots infected by RKNs assigned to regulation category by MapMan at (**A**) 7 and (**B**) 14 dpi.

Among the 5360 core genes, ARF TFs were the most abundant (22), followed by HSP (14), ERF (13), MYB (11), bHLH (9), WRKY (9), and NAC (6) (Table S4). Most of these TFs were induced by the RKN infection. WRKY TFs constitute a large family of transcriptional regulators that respond to biotic stress, particularly disease resistance. In our study, 9 WRKY TFs were identified among the different stages after RKN infection (Figure 6 and Table S4). Using MapMan, DEGs at 7 and 14 dpi were classified into different processes. Many hormone signaling pathways (auxin, BR, ABA, and SA), redox signaling, transcription factors (ERF, WRKY, and MYB), and HSPs were involved in the response to biotic stress (Figure 7).

**Figure 8:**
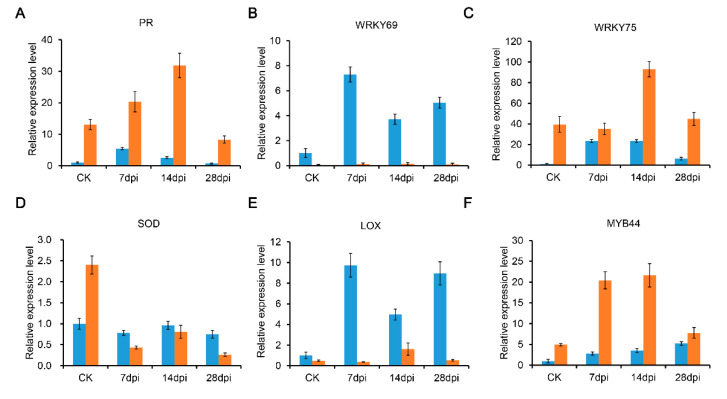
Validation of the expression levels of some differentially expressed genes (DEGs) in Sme and Tor using qRT-PCR: (**A**) PR; (**B**) WRKY69; (**C**) WRKY75; (**D**) SOD; (**E**) LOX; and (**F**) MYB44. The blue column represents Sme sample, and the orange column represents Tor sample.

To confirm the DEGs identified from the RNA-seq data between the root samples of Sme and Tor in the four stages, six DEGs related to the RKNs resistance, including PR gene, WRKY, and MYB, were selected for qRT-PCR analysis (Figure 8). These genes showed different expression patterns, consistent with those obtained from the RNA-Seq between Sme and Tor.

## 3. Discussion

As an important vegetable crop, eggplant is severely threatened by abiotic and biotic stresses, especially root-knot nematodes (RKNs). The wild relatives of eggplant, such as *Solanum torvum* (Tor), have been documented to be resistant to RKNs [18,23]. Identification of the genes for Tor resistance could be valuable for cultivated eggplants [18,23]). However, few studies have reported the molecular mechanisms of the defense responses of eggplant to RKNs. In this study, we attempted to shed more light on the issue by performing root transcriptome analysis of eggplant infected with RKN Meloidogyne incognita, comparing the transcription profiling of Tor with that of Sme. 

Generally, differential expression changes in genes involved in stress and defense response, metabolism, the cell wall, and signal transduction respond to nematode infection in plants [24,25,26]. In the transcriptome analysis, 8148 and 4761 DEGs were induced in Sme and Tor in all the stages under RKN infection (Figure 2B,C). A total of 5360 DEGs were identified between Sme and Tor among the four stages, suggesting that these genes might play important roles in the resistance of eggplant to RKN (Figure 3). GO and KEGG enrichment analysis showed that response to stimulus, response to hormone, cell wall biogenesis/organization, plant hormone signal transduction, and plant–pathogen interaction were overrepresented (Figure 2 and Figure 5). Nitrogen compound metabolic process and transport were enriched at 7 and 14 dpi compared with CK, implying that nutrient transport might be important in these stages (Figure 2D). In addition, the metabolic activity of roots might be inhibited after RKN infection, and many DEGs were enriched in different metabolism pathways (Figure 5A).

Due to the antimicrobial activity, PRs are well-known in the response to biotic stress [27]. The presence of a large number of PRs in the susceptible line Sme compared with the resistant Tor might be due to the basal defense responses (Figure 3D). Nucleotide-binding site–leucine-rich repeat (NBS-LRR) is a family of resistance genes that respond to biotic stress in many plants [28,29]. These R genes contain two typical domains: NBS and C-terminus LRR, which is an effector-binding domain that recognizes the pathogen effector molecules [23,30]. NBS-LRR motifs are widely found in nematode R genes in plants [28]. In tomato, one R gene, Mi-1.2, which belongs to the NBS-LRR class, has been reported to confer resistance against three species of Meloidogyne [31]. In our study, we identified 24 DEGs of NBS-LRR R genes (Table 1 and Table S4). Most of these R genes were up-regulated in Sme, suggesting that they are associated with RKNs resistance in eggplant.

Calcium signaling plays a key role in the plant response to environmental change. Previous research demonstrated calcium/calmodulin-mediated defense signaling is a key mechanism for nematode resistance in wild *Glycine soja* [32]. Our study also revealed that many DEGs encoding calcium-dependent protein kinase are highly expressed in the wild relative of eggplant, Tor (Table 2).

In plants, the cell wall is the first structural barrier to infection by many pathogens [23]. In the present study, some DEGs were related to the response to wound, chitin and lignin catabolic process (Figure 7 and Table 3). Dozens of genes participated in cell wall were up-regulated in Sme, indicating it may be a defense strategy. Xyloglucan endotransglucosylases (XETs) are enzymes that have specific activity on xyloglucans. Additionally, the structural changes in cell wall xyloglucans are associated with cell expansion, which is essential for nematode feeding sites formation [23]. Our study showed that many XETs (Sme2.5_00135.1_g00019.1, Sme2.5_00096.1_g00018.1) were repressed in Tor by RKN infection (Table 3).

Phytohormones such as SA, GA, and ABA play important roles in the regulation of the defense responses [33,34,35]. SA and GA signaling pathways have been reported to be mutually antagonistic, while the ABA has mostly been considered to negatively regulate disease resistance [36]. In Arabidopsis, SSI4 which encoded a R protein of the TIR-NBS-LRR class was induced by SA [36,37]. In the present study, DEGs encoding four SA and eleven GA-related genes were identified (Table S5). Interestingly, many ABA-related genes were differentially expressed between Sme and Tor (Table S6). The *NCED* is thought to be a key enzyme in ABA biosynthesis and the expression level of one *NCED* gene was up-regulated in Tor (Figure 5B and Figure S5). Additionally, two abscisic acid hydroxylase (ABH) genes were also differential expression (Table S6). These results suggest that the ABA signaling pathway was related to the response to RKNs in eggplant. In addition, BR biosynthesis gene BAK1 is highly expressed in Tor. The BAK1 and FLS2 genes play important roles in the plant immune response, and the RKN effectors may interact with BAK1 an FLS2 during invasions [16]. Thus, BR might be important for the defense against RKNs.

MapMan mapping also showed the transcription factors (ERF, MYB, WRKY, and HSP) involved in the defense against RKNs (Figure 6 and Figure 7). Previous studies reported that ethylene signaling can positively regulate the plant response to RKN invasion via activating jasmonate biosynthesis [24,38]. In our study, a large number of ERFs were differentially expressed in Sme and Tor (Figure 6 and Table S5). WRKY is a large family of transcriptional regulators in plant–pathogen interactions. Previous studies reported that WRKY23 influences the early response to RKN infection [7]. We also identified many WRKYs that were differentially expressed between Sme and Tor (Table S4). The expression levels of WRKY69 and WRKY75 were validated by qRT-PCR (Figure 8). Therefore, the WRKY genes are involved in the pathogen resistance of the eggplant plant host. In addition, NAC TFs have been reported to regulate cell wall remodeling after RKN infection in Cucurbitaceae crops [7].

## 4. Materials and Methods

### 4.1. Plant Material and Nematode Infection

Seeds of *Solanum torvum* Sw accession TG1 (RKN-resistant) and eggplant (*Solanum melongena* L., RKN-susceptible) breeding line Sme were sown in a seed plot for germination according to Gousset et al. (2005) [39]. Seedlings at the fifth-leaf stage were transplanted into 15 cm diameter plastic pots, with 500 cc of a mixture of sterilized sandy soil [40]. The pots were maintained in controlled chambers at 28 °C, 70% humidity, with a 14 h light/10 h dark. Then, the resistant (torvum (Tor)) and susceptible (Sme) lines were inoculated at the base of the plant stem with hatched root-knot nematode (Meloidogyne incognita, RKN) at the second larval stage (J2). RKNs were maintained in the greenhouse on eggplant; eggs masses were extracted as described by Shukla et al. (2018) [19]. For transcriptome study, the Sme and Tor lines were sampled at the uninfected stage (CK) and 7, 14, and 28 days post-infection (dpi) in the susceptible response. The soil was removed from the root tissues collected from each type of plant, the plants were washed with water and then frozen in liquid nitrogen to prevent RNA degradation. Finally, root samples were stored at –80 °C until RNA isolation.

### 4.2. RNA Extraction and Library Preparation for Transcriptome Sequencing

Total RNA was extracted from *S. torvum* (Tor) and eggplant (Sme) roots with three biological replicates for each condition using the Trizol RNA extraction Kit (Tiangen, Beijing, China). RNA quality was assessed using an Agilent Bioanalyzer 2100 (Agilent Technologies, Foster, CA, USA). In all the samples tested, the RNA integrity number (RIN) was above 9, whereas the concentration ranged from 200 to 300 ng/μL. Then, 1μg of RNA per sample was used as the input material for RNA sample preparation. Sequencing libraries were produced using NEB ultra RNA Library Prep Kit for Illumina (NEB, Columbia, MA, USA) following the manufacturer’s recommendations. The quality of the PCR libraries was assessed on an Agilent Bioanalyzer 2100 system (Agilent Technologies, Foster, CA, USA). Finally, PCR libraries were sequenced on an Illumina Hiseq4000 platform and 150 bp paired-end reads were generated. All the clean reads were deposited in the National Center for Biotechnology Information (NCBI) Sequence Read Archive under accession: PRJNA688588.

### 4.3. De Novo Assembly of S. torvum Transcriptome and Functional Annotation

Using Trinity, the clean reads of Tor samples were assembled in transcripts and unigenes [41]. Then, the sequences of these unigenes were further searched against to four protein databases by BLASTx with E-value < 1 × 10^−5^. The databases included NCBI non-redundant protein (NR) database, NCBI nucleotide (NT) database, Pfam, Clusters of eukaryotic Orthologous Groups of proteins (KOG) database, Swiss-Prot, Gene Ontology (GO) and Kyoto Encyclopedia of Genes and Genomes (KEGG) pathway database. Moreover, the homologous genes of Tor for eggplant were identified using BLASTn. FPKM (fragments per kilobases of exon per million fragments mapped) values for each sample were counted using RSEM software [42].

### 4.4. Identification of Differentially Expressed Genes 

After sequencing, the raw data, in FASTQ format, were filtered through the FASTX-Toolkit. The clean reads were obtained by trimming the adapter contaminants, removing low-quality reads, and reads containing poly-N. Q20 and Q30 scores and the GC content of the clean data were calculated by FastaQC. All the downstream analyses were based on high-quality clean reads. TopHat2 (version 2.1.0) was used to map these paired-end clean reads to the Eggplant Genome Database reference genome (http://eggplant.kazusa.or.jp/, accessed on 1 October 2014) [43]. Transcript assembly was conducted using Cufflinks software version 2.2.1 [44]. DESeq2 was used to identify DEGs with the fragments per kilobases of exon per million fragments mapped (FPKM) values [45]. The significant difference was determined with a false discovery rate (FDR) threshold of 0.05 and log2 fold change of ≥1 or ≤−1.

### 4.5. Gene Ontology (GO) and Kyoto Encyclopedia of Genes and Genomes (KEGG) Pathway Enrichment Analysis

To assign the function annotation, all the assembled transcripts were searched using Blastx (E-value < 1 × 10^−5^) against Pfam (http://pfam.janelia.org/, accessed on 1 October 2020), Swiss-prot (UniProt), and NR (NCBI). The DEGs were annotated with Gene Ontology (GO) terms to investigate the putative functions. Additionally, GO enrichment analysis was performed using the AgriGO program [46]. The statistical significance of GO terms was measured by a Fisher’s exact test corrected by an FDR of <0.05. The Kyoto Encyclopedia of Genes and Genomes (KEGG) pathways were determined using KOBAS3.0 (http://kobas.cbi.pku.edu.cn/, accessed on 1 October 2020) with an enrichment *p*-value < 0.05 [47]. Moreover, the differentially modulated genes between Sem and Tor at 7 dpi and 14 dpi were mapped using MapMan v3.6.0. Additionally, the transcript regulation categories were classified.

### 4.6. Quantitative Real-Time PCR (qRT-PCR) Analysis

Total RNA was extracted from the roots of Sme samples at four stage using Trizol (Invitrogen, Carlsbad CA, USA) according to the manufacturer’s instructions. Approximately 2 μg of total RNA was reverse transcribed using a RevertAid First Strand cDNA Synthesis Kit (Fermentas, Waltham, MA, USA). Then, the cDNA was synthesized and qRT-PCR was performed in 20 μL of reaction mixture with the ABI One step plus Real-Time Detection System (Applied Biosystems, Foster, CA, USA) using the Toybo SYBR Mix with ROX (CWBIO, Beijing, China). The thermal profile for qPCR was 95 °C for 3 min, followed by 45 cycles of 95 °C for 15 s, and 60 °C for 1 min. Three biological replicates were performed for each sample. All the primers used in this study are listed in Table S1. A melting curve analysis was performed to determine the specificity of the products. The reference gene (tubulin gamma (Sme2.5_03686.1_g00005.1)) was used for normalization. The relative gene expression level for each gene was analyzed using the comparative 2^△△CT^ method [48].

## 5. Conclusions

In this study, we investigated the transcriptome profiles of eggplant and its relative species and the defense mechanisms against RKN invasion. In total, we identified 9214 DEGs in response to RKN infection at four time points (ck, 7, 14, and 28 dpi). GO and KEGG enrichment analysis revealed that these DEGs are mainly involved in hormone signal transduction and plant-pathogen interaction pathways. Many pathogen-associated genes and NBS-LRR genes were induced during the RKN infection. Some plant hormone (auxin, ethylene, and ABA)-related genes were also differentially expressed, indicating their roles in the defense resistance. Our findings are important for further research on the defense mechanisms of eggplant against RKNs invasion. Furthermore, the transcription factors and ABA signaling genes identified in our study might be useful for developing nematode-resistant cultivars in eggplant breeding.

## Figures and Tables

**Table 1:** Some R genes identified for disease resistance of RKN in eggplant.

Gene ID	Alias	CK ^a^	7 dpi	14 dpi	28 dpi	Annotation
Sme2.5_00077.1_g00005.1	*RPP13*	1.73	1.13	0.68	1.58	Disease resistance protein RPP13
Sme2.5_03872.1_g00001.1	*RGA1*	0.61	0.32	0.63	0.37	Disease resistance protein RGA1
Sme2.5_03872.1_g00002.1	*RGA4*	0.39	0.25	0.21	0.26	Disease resistance protein RGA4
Sme2.5_00130.1_g00024.1	*RPM1*	0.38	1.47	0.13	0.06	Disease resistance protein RPM1
Sme2.5_00345.1_g00018.1	*RPP8*	0.37	0.84	0.64	0.33	Disease resistance protein RPP8
Sme2.5_07918.1_g00005.1	*RPM1*	7.79	2.74	3.66	3.23	Disease resistance protein RPM1
Sme2.5_02954.1_g00001.1	*RPV1*	0.98	2.20	3.86	2.65	Disease resistance protein RPV1
Sme2.5_05922.1_g00005.1	*RPM1*	0.07	0.09	0.03	0.10	Disease resistance RPM1
Sme2.5_10927.1_g00001.1	*RGA4*	7.92	4.52	5.64	6.58	Disease resistance protein RGA4
Sme2.5_06008.1_g00004.1	*RGA3*	0.56	0.10	0.08	0.10	Disease resistance protein RGA3
Sme2.5_16574.1_g00002.1	*R13L4*	0.58	0.87	1.20	1.34	Disease resistance RPP13-like protein 4
Sme2.5_13369.1_g00002.1	*RGA4*	0.20	0.28	0.11	0.26	Disease resistance protein RGA4
Sme2.5_19803.1_g00001.1	*R13L4*	0.88	0.29	0.27	1.14	Disease resistance RPP13-like protein 4

^a^ The relative expression between Sme and Tor, FPKM value (Sme)/FPKM value (Tor).

**Table 2:** Expression patterns of plant-pathogen interaction pathway genes in Sme and Tor.

Gene ID	Alias	CK ^a^	7 dpi	14 dpi	28 dpi	Annotation
Sme2.5_00057.1_g00028.1	*PR1*	2.651	1.797	1.175	1.868	Pathogenesis-related protein 1
Sme2.5_05922.1_g00005.1	*RPM1*	0.071	0.093	0.028	0.096	Disease resistance protein RPM1
Sme2.5_03682.1_g00009.1	*DRL27*	0.258	0.047	0.144	0.292	Disease resistance protein DRL27
Sme2.5_09833.1_g00003.1	*GSO2*	0.865	4.465	0.425	0.399	LRR receptor-like serine/threonine-
						protein kinase GSO2
Sme2.5_00365.1_g00011.1	*CML16*	0.135	0.140	0.169	0.100	Calcium-binding protein CML16
Sme2.5_02438.1_g00002.1	*CDPK8*	0.238	0.222	0.224	0.435	Calcium-dependent protein kinase 8
Sme2.5_00959.1_g00002.1	*CDPK2*	3.369	0.940	0.942	1.091	Calcium-dependent protein kinase 2
Sme2.5_00572.1_g00003.1	*CNG18*	0.304	0.340	0.241	0.289	Cyclic nucleotide-gated ion channel 18
Sme2.5_00629.1_g00003.1	*MRK7*	5.936	12.89	8.233	9.426	Mitogen-activated protein kinase kinase 7
Sme2.5_00861.1_g00010.1	*MRK3*	3.917	4.601	5.035	7.191	Mitogen-activated protein kinase kinase 3
Sme2.5_01071.1_g00003.1	*WRKY22*	1.240	2.136	2.084	2.632	WRKY transcription factor 22
Sme2.5_00297.1_g00009.1	*HSP90*	1.240	2.136	2.084	2.632	Heat shock 90 kDa protein

^a^ The relative expression between Sme and Tor, FPKM value (Sme)/FPKM value (Tor).

**Table 3:** Expression patterns of genes associated with cell wall in Sme and Tor.

Gene ID	CK ^a^	7 dpi	14 dpi	28 dpi	Annotation
Sme2.5_00236.1_g00007.1	6.104	1.235	1.871	2.275	Chitin-inducible gibberellin-responsive protein
Sme2.5_18829.1_g00001.1	111.4	72.32	4.060	2.670	Basic endochitinase A
Sme2.5_03222.1_g00007.1	11.86	65.26	26.70	74.93	Endochitinase 1
Sme2.5_00287.1_g00007.1	1.346	16.19	2.376	3.592	Wound-induced protein
Sme2.5_03391.1_g00004.1	1.911	5.025	2.275	0.807	Lignin-forming anionic peroxidase
Sme2.5_03391.1_g00006.1	3.010	2.131	3.572	1.007	Lignin-forming anionic peroxidase
Sme2.5_00295.1_g00005.1	0.679	0.189	0.026	0.394	Laccase-3
Sme2.5_00135.1_g00019.1	7.591	2.562	2.642	2.476	Xyloglucan endotransglucosylase/hydrolase 16
Sme2.5_03583.1_g00006.1	1.745	1.184	0.443	0.226	Xyloglucan endotransglucosylase/hydrolase 2
Sme2.5_00498.1_g00013.1	0.031	0.052	0.410	0.000	Xyloglucan endotransglucosylase/hydrolase 26
Sme2.5_00096.1_g00018.1	8.966	1.924	1.952	2.720	Xyloglucan endotransglucosylase/hydrolase 25

^a^ The relative expression between Sme and Tor, FPKM value (Sme)/FPKM value (Tor).

## Data Availability

Not applicable.

## Supplementary Materials

The following are available online at https://www.mdpi.com/article/10.3390/pathogens10040470/s1, Figure S1: Sample correlation between the 24 samples. Figure S2: Detailed information if the transcriptome assemblies of Tor. Figure S3: Schematic of the plant-pathogen interaction pathway category from the Kyoto Encyclopedia of Genes and Genomes (KEGG). Figure S4: Heatmap showing the expression patterns of genes encoding F-box/LRR-repeat and TMV resistance protein. Figure S5: The relative expression of genes involved in the abscisic acid (ABA) metabolism pathway. Table S2: Summary of the sequence data generated by RNA-Seq. Table S2: Summary of the transcriptome assemblies of Tor. Table S3 Detailed information of simple sequence repeat (SSR) and primers in Tor. TableS4: The expression patterns of the 5360 differentially expressed core genes for root-knot nematode (RKN) infection. Table S5: The expression patterns of 4020 differentially expressed genes for resistance to RKNs. Table S6: The expression patterns of differentially expressed genes related to phytohormones and transcription factors. Table S7: KEGG pathways of differentially expressed genes between Sme and Tor. Table S8: Primers used for qRT-PCR in the study.
