# Supplementary figures and images for: Transcriptome Analysis of Eggplant Root in Response to Root-Knot Nematode Infection
Source: Pathogens. 2021 Apr 13;10(4):470. doi: 10.3390/pathogens10040470 (PMC8069755; doi:10.3390/pathogens10040470)

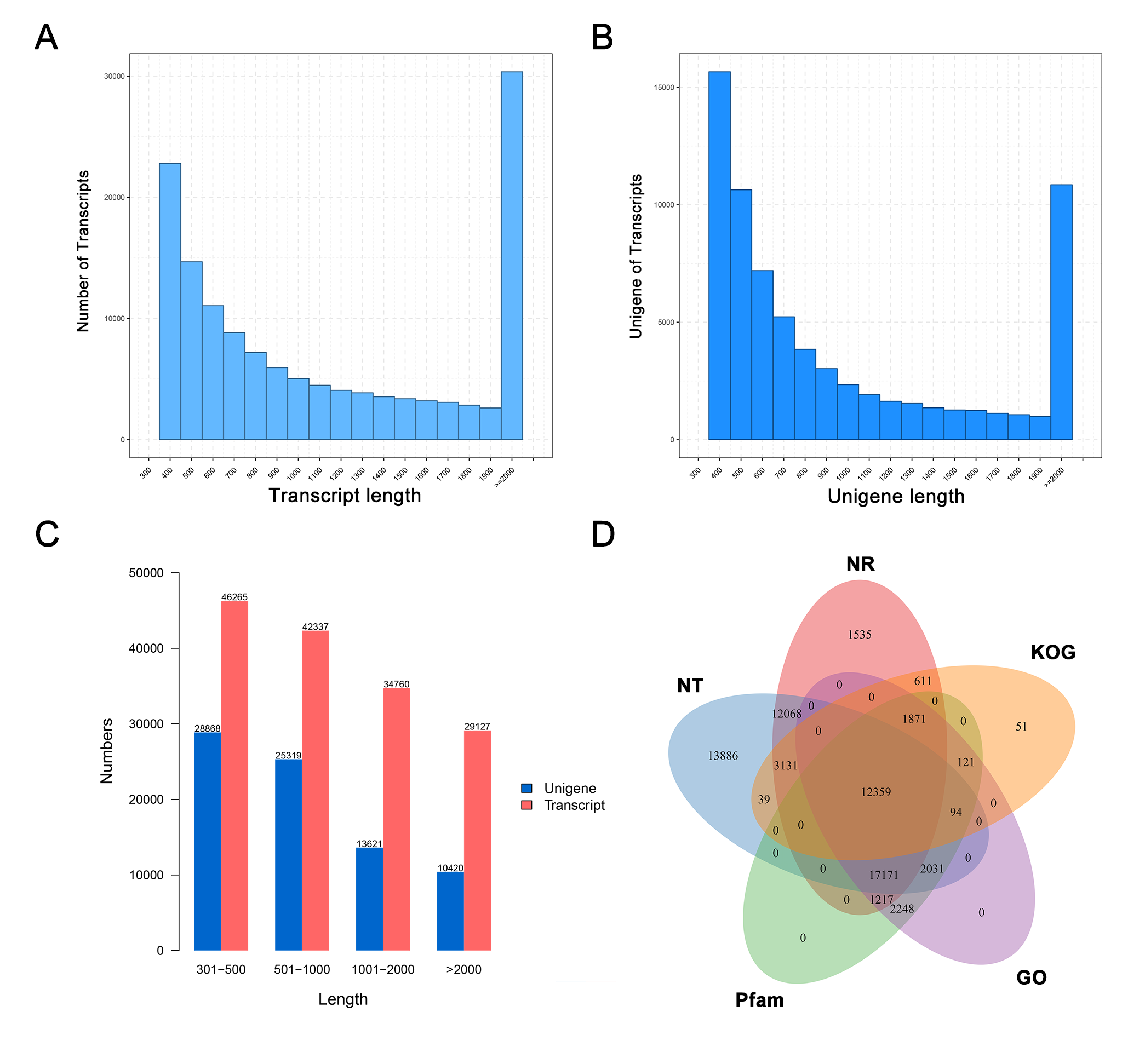

Supplement: Supplementary file 1 [file pathogens-10-00470-s001.zip › Figure S2.tif]

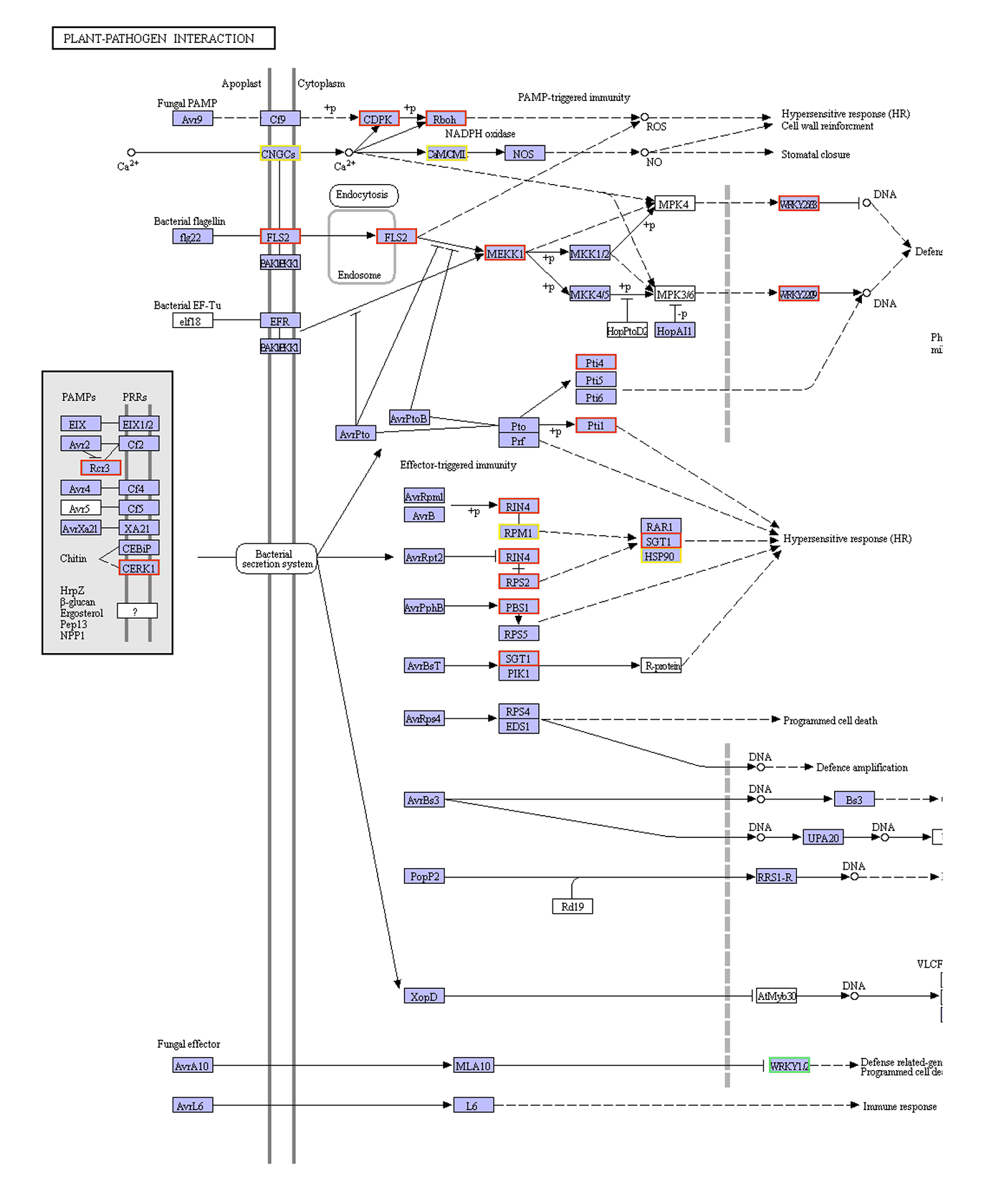

Supplement: Supplementary file 1 [file pathogens-10-00470-s001.zip › Figure S3.tif]

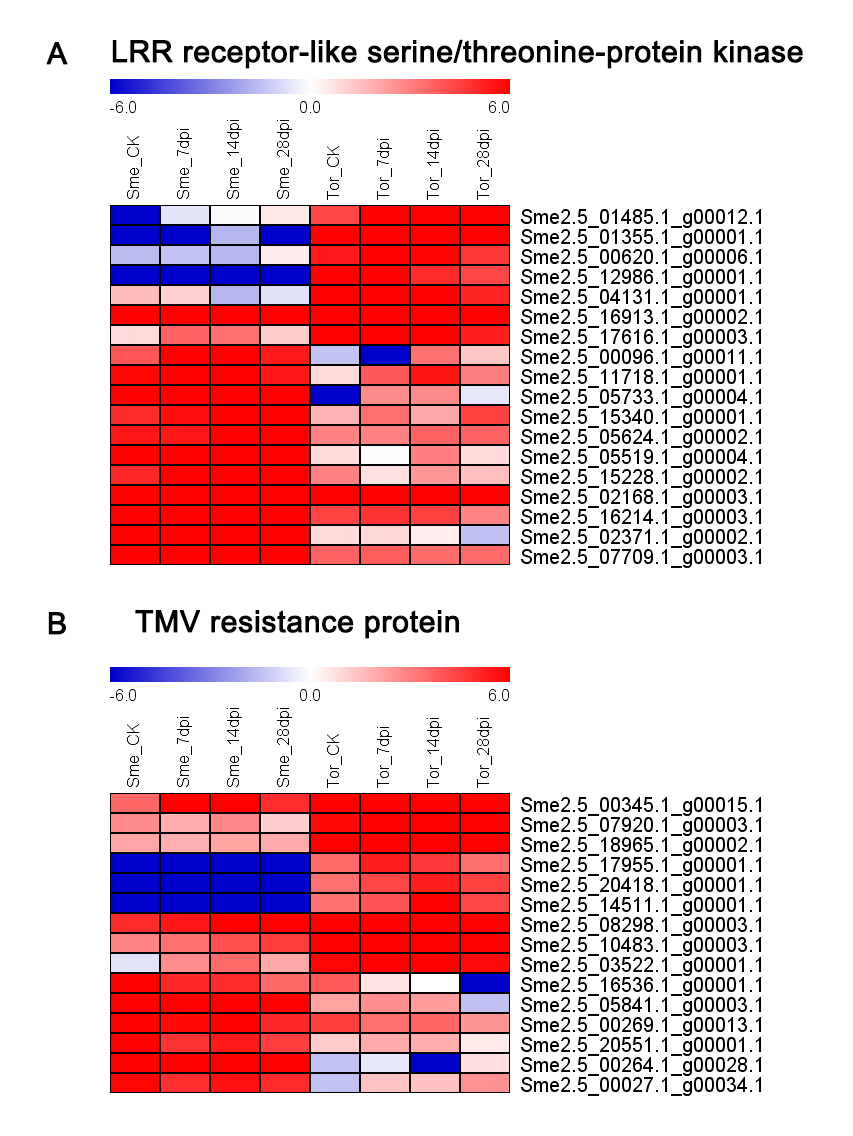

Supplement: Supplementary file 1 [file pathogens-10-00470-s001.zip › Figure S4.tif]

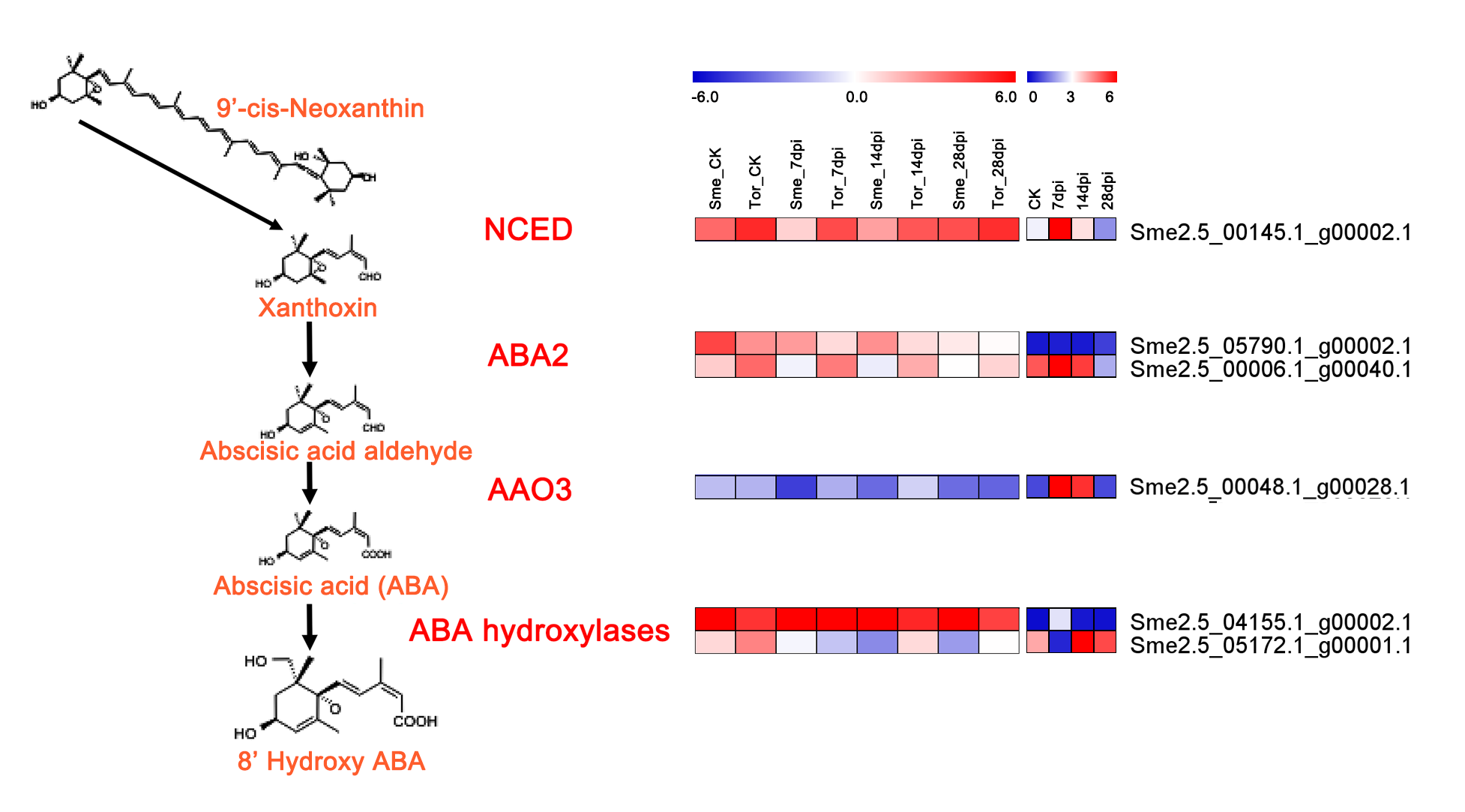

Supplement: Supplementary file 1 [file pathogens-10-00470-s001.zip › Figure S5.tif]

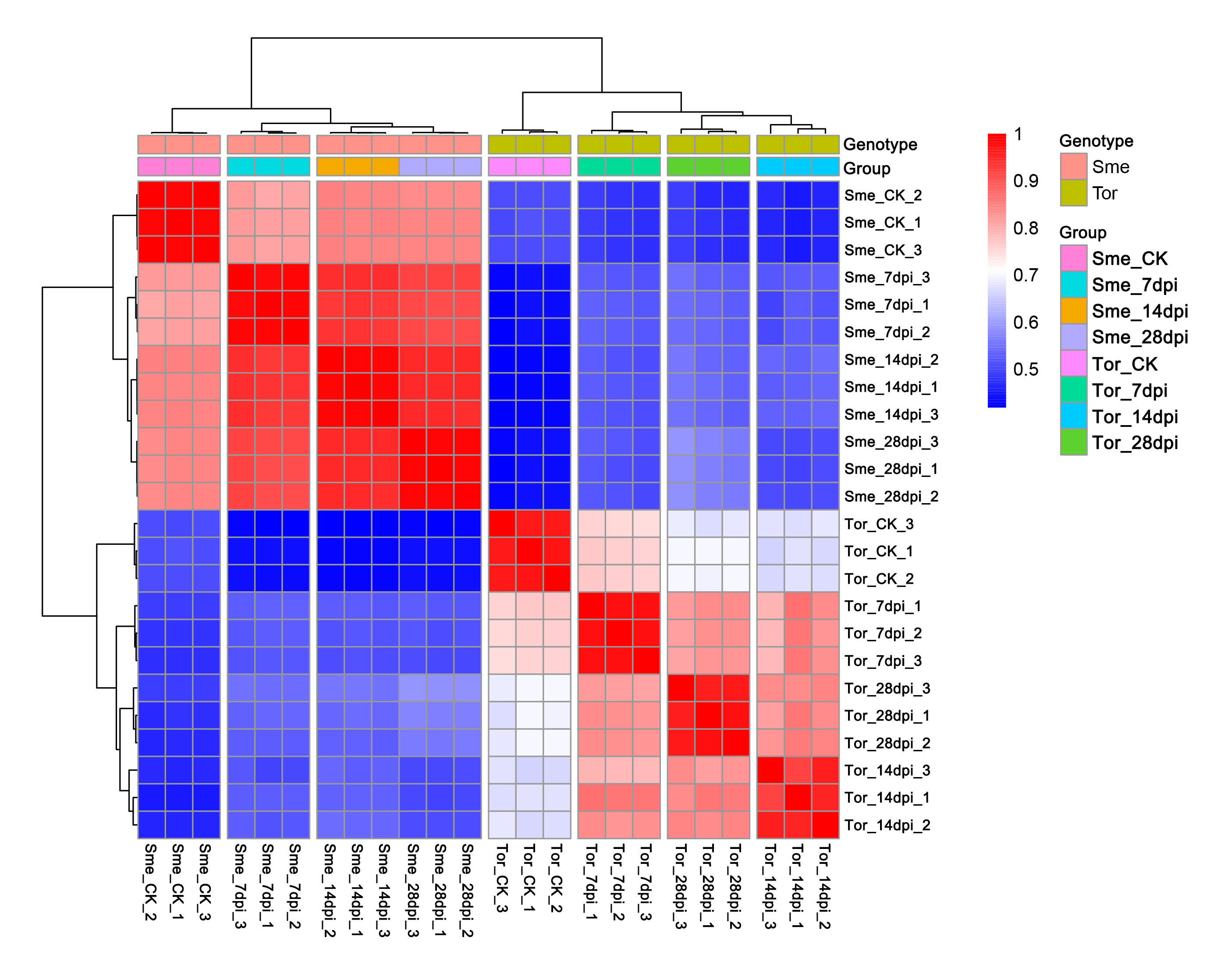

Supplement: Supplementary file 1 [file pathogens-10-00470-s001.zip › Figure S1.tif]
